# Geographical Variation in Oral and Oropharynx Cancer Mortality in Brazil: A Bayesian Approach

**DOI:** 10.3390/ijerph15122641

**Published:** 2018-11-25

**Authors:** Emílio Prado da Fonseca, Regiane Cristina do Amaral, Antonio Carlos Pereira, Carla Martins Rocha, Marc Tennant

**Affiliations:** 1 Health Surveillance Department, Divinópolis, Minas Gerais 35500-007, Brazil; 2 Dentistry Department, Federal University of Sergipe, Aracaju, Sergipe 49060-108, Brazil; amaralre@yahoo.com.br; 3 Department of Community Dentistry, Preventive Dentistry and Public Health area of Piracicaba Dental School, FOP/UNICAMP, University of Campinas, Piracicaba, São Paulo 13414-903, Brazil; apereira111@gmail.com; 4 International Research Collaborative-Oral Health Equity Anatomy, Physiology and Human Biology, University of Western Australia, Perth 6907, Australia; c.mrocha@hotmail.com (C.M.R.); marc@ircohe.net (M.T.)

**Keywords:** spatial, epidemiology, oral cancer, mortality, health geography

## Abstract

Recent studies have shown a high number of deaths from oral and oropharyngeal cancer worldwide, Brazil included. For this study, the deaths data (ICD-10, chapter II, categories C00 to C14) was obtained from Mortality Information System (SIM) and standardized by gender and population for each of the 554 Microregions of Brazil. The raw mortality rates were adopted as the standard and compared to the application of smoothing by the Bayesian model. In order to describe the geographical pattern of the occurrence of oral cancer, thematic maps were constructed, based on the distributions of mortality rates for Microregions and gender. *Results*: There were 7882 deaths registered due to oral and oropharyngeal cancer in Brazil, of which 6291 (79.81%) were male and 1591 (20.19%) female. The Empirical Bayesian Model presented greater scattering with mosaic appearance throughout the country, depicting high rates in Southeast and South regions interpolated with geographic voids of low rates in Midwest and North regions. For males, it was possible to identify expressive clusters in the Southeast and South regions. *Conclusion*: The Empirical Bayesian Model allowed an alternative interpretation of the oral and oropharynx cancer mortality mapping in Brazil.

## 1. Introduction

Each year the number of cancer deaths increase worldwide [1,2,3]. In 2012, 145,000 deaths were registered due to oral cavity and oropharynx cancer, of which 77% were in the less developed regions [2]. Oropharynx cancer etiology is multifactorial, with the main risk factors being tobacco, alcohol, mechanical trauma, biological agents, genetic predisposition, individual’s systemic status, and diet. Mortality rates for these neoplasms are associated with late diagnosis [4].

Historically, the incidence of oral cavity and pharyngeal cancers has been highest in South and South-East Asia, Western and Central Europe, and South America [2]. However, a recent study predicted that more than 10,000 deaths from oral cavity and pharynx cancer are expected for 2018 in the US [5]. The mortality rates in South American countries ranges from 0.72% to 6.04% per 100,000 population, and the proportion of ill-defined deaths in South America varied from 5.0% to 22.0% [6]. Mortality trends for males decreased about 2.5% in most of the countries, excluding Brazil, whereas among females, a significant decrease occurred only in Colombia, with an increase in Brazil and Peru [6]. Between 2002 and 2013, 74,342 deaths from oral and pharyngeal cancer were registered in Brazil, corresponding to 3.9% of the deaths from all neoplasms in the period studied [7].

Mapping is a widely used epidemiology tool, often employed as the first step to define an epidemic, to visualize spatial distribution, and to indicate areas of high occurrence or predominance of the event [8]. Consistent with the temporal heterogeneity in the prevalence worldwide, oral and pharyngeal cancer mortality show wide variability by geographic region and era [1,2,5,8]. Most epidemiological studies of oral cancer use a crude and gender-age-weighted rate to analyze the findings [2,5,6,7].

Bayesian estimation is a mathematical smoothing method used to improve the accuracy of rates of events that would otherwise be equal to zero in regions with small populations, or areas presenting with few or no events. This methodology has been previously applied to the study of other types of cancer [9,10,11,12]. Bayesian inference could, therefore, be used to smooth mortality rates and enable better visualization and interpretation of the distribution of deaths from oral and oropharynx cancer [13]. The aim of this study was to compare the results obtained between the estimate by the crude rate and Bayesian estimation from oral and oropharynx cancer mortality in Brazil in 2016.

## 2. Materials and Methods

Data were obtained from the Mortality Information System (SIM) available in the website of the Information System of the Brazilian Unified Health System (DATASUS) [14]. Specifically, the number of deaths attributed to oral and oropharynx cancer (categories C00 to C14), according to the International Classification of Diseases, Chapter II, Tenth Revision (ICD-10) were collected and standardized by gender and population for each of the 554 Microregions of Brazil. Four (0.05%) deaths were excluded from this study due to the lack of information regarding location and/or gender. Population estimates for the year 2015 was provided by the Brazilian Institute of Geography and Statistics (IBGE) [15]. 

The spatial unit used to aggregate the data was the Brazilian ‘Microregions’, defined by the IBGE as regions constituted by groups of adjacent municipalities to attend the basic needs of the population, including a combination of private and public services providing education, health, jobs, etc. The geographic limits of the Microregions are updated periodically (usually every decade) and made available at the IBGE website: https://www.ibge.gov.br/apps/regioes_geograficas/. The Raw Rates of Oral and Oropharynx Cancer Mortality (OOCM) were calculated by the number of deaths in each Microregion (*n* = 554) divided by the population at risk and later multiplied by 100,000 inhabitants and weighted by gender [13]. These raw rates were adopted as the standard for comparison with the application of smoothing by the Bayesian model [11,13]. The Empirical Bayesian Rate (EBR) was defined by the pondered sum between raw rate and the overall/global mean rate [13]. Empirical Bayesian smoothing leaves estimates for areas with low margins of error alone, but nudges estimates in regions with high margins of error closer to the overall average of the event rate [13]. Ponderation has a factor inversely proportional to that of the population exposed [11,13]. The first step of data analysis was composed of prevalence calculations and measures of central and dispersion measures [16].

Univariate exploratory analysis of EBR and weighted gender were performed for the correlation of a variable with itself throughout the space [16]. The phenomenon, in which areas with similar values are distributed in a non-random way, is called spatial autocorrelation [16]. Substantially, this is consistent with the expectation that the regions will gather spatially (formation), and more importantly, in such a way that oral and oropharynx cancer mortality has spatial dependence [16]. In this sense, spatial weights are a key component in any cross-sectional analysis of spatial dependence and construction of spatial autocorrelation statistics [16]. The core input into the determination of a neighbor relation for distance-based spatial weights is a formal measure of distance or a distance metric [16]. Then, the neighborhood matrix was calculated using the Queen Contiguity criterion.

Spatial autocorrelation analysis was used to demonstrate and explain the existing patterns of spatial association (clustering) of deaths distribution of oral and oropharynx mortality among Brazilian regions [16,17]. The investigation of EBR pattern was performed through the Global Moran Index (I) [16,17]. The (I) indicates the degree of spatial association of a variable with respect to the data set [16]. Moran’s I varies between −1.0 and +1.0 and is similar, but not equivalent, to a correlation coefficient [17]. Positive spatial autocorrelation occurs when similar values occur near one another [17]; negative spatial autocorrelation occurs when dissimilar values occur near one another and when I equals zero, there is no spatial autocorrelation [16,17]. For the validation of Moran’s I we used the test of 999 random permutations [17]. In order to evaluate the significance (*p* ≤ 0.05) of the Moran’s I, the following hypotheses are established: H0: I = 0 (there is no spatial self-relation between regions); and H1: I > 0 (there is positive spatial self-relationship between regions) [17].

Once verified, the significance of spatial autocorrelation by Moran’s I, the patterns of distribution was analyzed using the Local Indicator of Spatial Association (LISA) [16,17]. Positive LISA values (0 to +1) indicate a direct correlation and negative values (0 to −1) inverse correlation [17]. Essentially, the autocorrelation map distinguishes between the following types of groups (statistically significant at a level of 0.05): (1) Maximum values (HH); (2) Minimum values (LL); (3) dissimilar values (crass errors) in that a feature of high value is surrounded by low values (HL); and (4) Crass errors in which a feature with low value is surrounded by high value features (LH) [17].

Bioestat^®^ version 5.0 software was used to carry out the statistics [18]. In order to describe the geographical pattern of the occurrence of oral cancer, thematic maps were constructed, based on the distributions of mortality rates for Microregions and gender. The legends of the maps were standardized into five extracts and equal intervals to facilitate visualization. A digital cartographic database (available at the IBGE website) and the public domain software GeoDa [18] (available from the Center for Spatial Data Sciences-University of Chicago) were used for map construction.

## 3. Results

In 2016, 7.882 deaths were registered due to oral and oropharyngeal cancer in Brazil, of which 6.291 (79.81%) were male and 1.591 (20.19%) female. Of the 554 Microregions surveyed, 34 (6.31%) did not register any deaths from oral and oropharyngeal cancer in 2016. The mortality proportion was 3.95 higher for men than for women.

Considering the total number of events in the 554 Microregions studied, the raw rate of oral and oropharynx cancer in Brazil was 3.60 deaths per 100,000 population. The weighted rates for males were higher when compared to females and the total Brazilian rate. The rates smoothed by the Empirical Bayesian model for Brazil and weighted by gender showed lower standard deviations and variance when compared to the raw rates. The coefficients of variation (CV) for raw rates showed values higher than 30%, indicating high dispersion and heterogeneity around the data mean. In this sense, the CV of Female Bayesian Empirical rate was the lowest CV and it affirmed that the data related to the women were more homogeneous when compared with the others. In the case of Moran’s I, the EBR values were higher when compared to OOCM rates. However, the EBR for females was not significant (*p* = 0.103). This indicated that there was spatial autocorrelation of OOCM rates (the chance of not having spatial autocorrelation was less than 0.1%) (Table 1).

**Figure 1:**
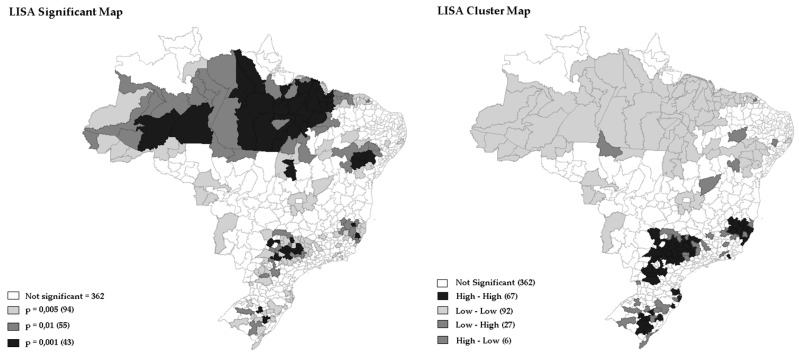
Univariate Local Moran’s by LISA of EBR from oral and oropharynx cancer, Brazil, 2016.

The identification of risk areas (spatial clusters with statistical significance), performed by LISA, confirmed that there is a spatial correlation of OOCM rate in Brazil, which cannot be explained by randomization. Figure 1 showed the degree of significance (*p*-value = 0.001 for 999 permutations) to the Univariate Local Moran’s by LISA of EBR of OOCM rate occurred in Brazilian regions in 2016. The analysis identified two large clusters, the first one involving areas from the North and Northeast regions, and the second covering part of the Midwest, Southeast and South regions. However, in 372 (65.34%) of the regions studied, there was no significance or spatial correlation by LISA. presented greater scattering with mosaic appearance. Deaths from oral cancer dispersed throughout the country with high rates in Southeast and South regions, interpolated with geographic voids of low rates represented by lighter colors in Midwest and North regions. Figure 1 depicts the redistribution of the raw rates proposed by the Empirical Bayesian Model, with the predominance of values represented by the intermediate layers in all the directions of the country. There was a reduction in the values of the lower layer of death rates, and increase in the middle rates, mostly in the North, Midwest, and Northeast (Figure 1).

**Figure 2:**
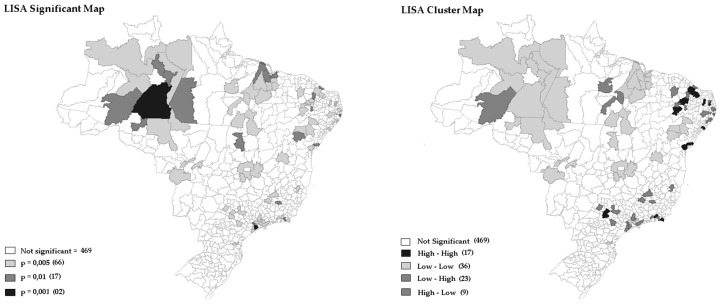
Univariate Local Moran’s by LISA of oral and oropharynx cancer weighted by female, Brazil, 2016.

**Figure 3:**
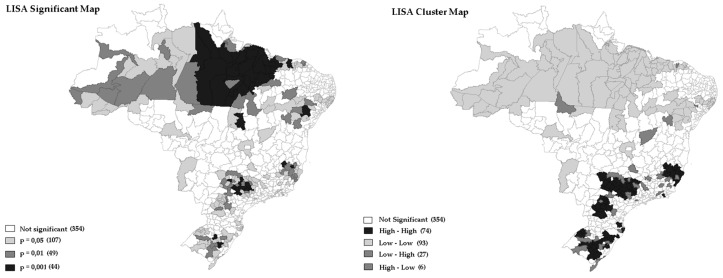
Univariate Local Moran’s by LISA of oral and oropharynx cancer weighted by male, Brazil, 2016.

Figure 2 and Figure 3 showed the degree of significance (*p*-value = 0.001 for 999 permutations) to the LISA of EBR weighted by gender of OOCM rate distribution of mortality rates from oral and oropharynx cancer that occurred in Brazilian regions in 2016.

Additionally, in Figure 1, Figure 2 and Figure 3 it was possible to observe the occurrence of spatial autocorrelations of the “High-High” cluster, highlighting the grouping of 67 (12.09%) Brazilian regions with higher mortality rates and their respective neighboring areas with high mortality rate values was due to oral cancer located in the following regions: Southeast, Midwest, and South. It was also observed the occurrence of “Low-Low” spatial autocorrelations, consisting of a group of 27 (4.87%) microregions with the lowest rates, located mainly in the North and Northeast regions. In fact, the strongly colored regions (High-High and Low-Low) are therefore those that contribute significantly to a positive global spatial autocorrelation outcome.

Figure 2 and Figure 3 represent EBR for females and males. It is important to note how the maps capture different death rate distributions for each gender. Typically, women have lower mortality rates when compared to men. It is possible to observe, with clarity, the extension of the regions with higher mortality in the South region, compared to the lower rates of the North.

In the large patch of regions with high mortality rates for males, it is possible to identify expressive clusters in Southeast and South regions (Figure 3).

## 4. Discussion

This study highlighted the high incidences of male mortality due to oral cancer in Brazil, as well as the spatial inequalities in its distribution, with clusters formation. The visualization of such distribution is paramount to the formulation of new hypotheses in the study of risk factors. In addition, the comparison of estimated oral cancer mortality crude rates to those generated by an empirical Bayesian model was demonstrated, providing an alternative visualization and interpretation of the data.

Local Cluster Analysis (LCA) can be used to detect hot spots of oral cancer mortality rates [16,19]. LISA statistics provides estimates disaggregated to the level of regions of the spatial analysis units, allowing assessment of dependency relationships in different areas regarding the presence of clusters and outliers [16,19]. This study applied spatial autocorrelation methods to determine the spatial clustering associated with a single variable (rate of mortality of oral cancer), classified into high or low mortality risk. Furthermore, spatial clustering techniques are essential for starting approach identification of risk map; formulate new hypotheses and monitoring in oral health [19]. Other studies have also successfully used LCA to identify clusters of low (LL) and high (HH) cancer mortality risk in various areas [16], with the authors suggesting that geographical arrangement of area units on a map can identify the degree of spatial clustering of oral cancer deaths. In this sense, the hot spots of oral cancer mortality obtained from this study analysis reveal significant indications to the risk factors of oral cancer deaths.

When studying rare events, researchers need to be cautious because of problems of under or overestimation of the rates may occur [20]. In regions in which no cases occurred, or which have very small populations, the crude rate would be zero or close to zero, therefore the appearance of a single case in these regions may significantly alter the crude rate [13,20]. According to Matangra et al. (2013), observational epidemiological studies conducted with Bayesian inference can be a very attractive alternative in case of “zero” cases compared to the classical approach [20].

In the present study, some Microregions with more than 11 million inhabitants, such as São Paulo and Rio de Janeiro, with the number of deaths exceeding 500, presented raw mortality rates ranging from 4.49 to 4.46. Meanwhile, in the Microregions of Jales and Seridó Paraibano, with population below 160 thousand inhabitants and less than 20 deaths, the highest raw death rates were registered (10.95 and 9.80, respectively). These facts compromised the estimation of the raw rate and showed the influence of population size on the results obtained (Figure 1, Figure 2 and Figure 3). Notably, there were no significant differences between raw rate and Bayesian model in populous regions (e.g., São Paulo, Rio de Janeiro, Belo Horizonte, Porto Alegre, and Salvador).

Traditionally, identification of clusters of high mortality rates from oral cancer has been made by means of frequencies and pondered rates by age or gender [1,2,3], however, Bayesian models for stabilizing or smoothing mortality rates from oral cancer and identification of clusters have also been published [13,21,22]. Indeed, an unknown part of the variation of the Crude Mortality Ratio (CMR) may occur due to geographically varying unobserved risk factors [22]. However, maps can be seriously misleading because the CMRs tend to be extreme in less populated regions [22]. One of the major goals of mortality maps is to identify unobserved risk factors through the geographical variation of the deaths cases [19,22]. The results of the present study suggest that the application of Bayesian model to mapping and the identification of death clusters of this type of cancer in Brazil is a useful tool. Nevertheless, it is important to affirm that the Bayesian rates do not substitute the raw and pondered rates, but produce a smoothing effect, facilitating and enhancing the visualization of clusters [21,22].

The analysis of geographic patterns of oral cancer mortality is complex concerning old and new risk factors, so the interpretation of maps must be done cautiously [8]. With the use of Bayesian Empirical Model (Male and Female), it was possible to identify clusters of deaths in regions known for having both the best and worst socioeconomic conditions (Figure 2 and Figure 3. Previous studies in Brazil, and abroad, observed a statistically significant association of poor socioeconomic condition and low Human Development Index (HDI) with spatial distribution of oral cancer mortality [23,24,25,26]. In addition, previous studies observed positive associations among the proportion of the population with dental appointment within last year, percentage of consumption of oils and fats, percentage of consumption of ready-to-eat foods and industrial mixtures and percentage of overweight adults with this type of cancer [26]. Recently, it has been reported that environmental exposure to heavy metals is an important risk factor for developing oral cancer, with studies on animals showing that chronic intake of chromium (Cr) could induce oral cancer [27]. Another spatial association between soil heavy metal content and oral cancer incidence and mortality has also been assessed in Taiwan [19,28]. Moreover, a study conducted in Brazil by Moi et al. (2018), showed the consumption of pesticides, and HPV contamination, positively correlated with mortality rates from oral cancer in adults [26].

Early diagnosis and immediate treatment of oropharynx cancer can significantly reduce the morbidity and consequently improves overall long-term survival rates [1,2]. Tobacco and alcohol consumption have geographic impact on oral cavity and pharynx cancer and these factors may explain the higher death rate from oral cancer in men, and great difference in deaths distribution between male and female [1,2,8,19]. The findings of the present study were similar to a study in Taiwan that identified significant differences between male and female spatial distributions of oral cancer [19]. This suggests that the in the spatial distribution of oral cancer deaths is due to spatial differences in the distribution of risk factors between males and females [19]. In Taiwan, the study showed an elevated mortality rate from oral cavity cancer for females clustered in aboriginal communities [19]. Aboriginal women in eastern Taiwan had a much higher prevalence of cigarette smoking, alcohol drinking, and betel nut chewing when compared to women in other areas [19]. While studies have shown that cancer mortality rates from the oral cavity are lower in women, there is evidence that oral cancer rates have increased for females in Sao Paulo, which represents an inversion of previous trends among genders in the city [29]. This finding warrants further investigation.

We acknowledge that the present study has a number of limitations. Firstly, being a cross-sectional study, it could not establish causality. Secondly, the use of secondary data presents a risk of bias information. Pourhoseingholi et al. (2012) stressed that cancer mortality analysis need reliable death registry systems that reports death statistics annually [20]. Additionally, the analysis of death statistics is subject to misclassification, a major problem in epidemiological analysis leading to biased estimates, and possibly causing the underestimation of health risks [20]. It is important to point out that the Mortality Information System, whilst being the most comprehensive set of data existent in Brazil, is unfortunately likely to be subject to under-registration, particularly in small and remote regions, or when the quality of the information is unknown. In this sense, epidemiological studies of spatial distribution based on maps are sensitive to the quality of the data available for the areas to be studied and healthcare policy makers should keep that in mind when planning actions and making decisions based on this sort of data.

## 5. Conclusions

The smoothing of raw rates through Empirical Bayesian Smoothing Model allowed an alternative interpretation of the oral and oropharynx cancer mortality mapping in Brazil. It highlighted the clustering formation of oral cancer mortality rates, suggesting the need to redirect Brazilian policies aimed at the combating potential etiological contributors to oral cancer cases and reduce deaths for this cancer type.

## Figures and Tables

**Table 1:** Descriptive statistics and Global Moran Index of OOCM rates in Brazil, 2016.

Rate	Mean	Median	SD ^1^	Variance	CV (%) ^2^	Minimum	Maximum	I ^3^	*p* ^4^
Raw rate of Brazil	3.60	3.43	2.11	4.43	58	0.00	10.95	0.402	0.001
EBR of Brazil	3.79	3.72	0.81	0.66	21	1.82	6.43	0.446	0.001
Raw rate of Female	1.48	1.24	1.46	2.19	98	0.00	14.58	0.121	0.001
EBR of Female	1.54	1.53	0.04	0.00	3	1.38	1.78	0.057	0.103
Raw rate of Male	5.74	5.30	3.68	13.57	64	0.00	17.99	0.421	0.001
EBR of Male	6.11	5.90	1.42	2.01	23	2.57	10.57	0.465	0.001

^1^ Standard Deviation. ^2^ Coefficient of Variation. ^3^ Global Moran Index. ^4^ *p*-value.
